# An Update on the Diagnosis and Management of Calcium Crystal Disease

**DOI:** 10.1007/s11926-023-01106-9

**Published:** 2023-05-30

**Authors:** Rachael Flood, John Stack, Geraldine McCarthy

**Affiliations:** 1 Mater Misericordiae University Hospital, Eccles Street, Dublin, Ireland; 2 School of Medicine, Mater Misericordiae University Hospital &, University College Dublin, Dublin, Ireland

**Keywords:** Calcium Crystal Disease, Calcium Crystals, BCP Crystals, Pseudogout, CPPD, Crystal Arthritis

## Abstract

**Purpose of Review:**

This article aims to review the challenges to diagnosis and management of calcium crystal deposition diseases and evaluate the literature published over the past 3 years.

**Recent Findings:**

The awaited development of classification criteria is an essential step in the progression of calcium crystal deposition disease clinical research. There have been recent improvements in the accuracy of imaging for the diagnosis of crystal deposition diseases with published definitions of characteristic features. Factors associated with acute flares of disease have been identified and an association with increased cardiovascular risk has been demonstrated. Targeted treatment options for calcium crystal diseases remain elusive. However, there have been advances in understanding the molecular mechanisms of disease revealing potential targets for future drug development.

**Summary:**

Calcium-crystal deposition diseases are increasing in incidence and prevalence as populations age and continue to associate with a high burden of disability. Despite this, calcium crystal deposition disease remains under-studied with a paucity of evidence-based treatment guidelines.

## Introduction

The two most common types of calcium-containing crystals associated with articular and peri-articular disorders are basic calcium phosphate (BCP) and calcium pyrophosphate dihydrate (CPP). These crystals differ in their composition, mechanism of formation, clinical associations and exert diverse effects on the cells within joint tissues.

CPP crystals are either rod or rhomboid shaped, measure 1–20 micromoles (μm) and are weakly birefringent under polarized light microscopy (PLM) [1]. Calcium pyrophosphate deposition disease (CPPD) is a clinical manifestation of CPP crystal deposition, an umbrella term that includes acute CPP crystal arthritis (also known as “pseudogout”), chronic CPP crystal inflammatory arthritis, and CPPD with osteoarthritis (OA) [2]. Acute CPP (“pseudogout) is a common cause of inflammatory mono- or polyarticular arthritis in the older person. The knee is most often involved and less often the wrist or ankle. Other presentations of chronic CPPD associated arthropathies include those which can mimic osteoarthritis (OA), rheumatoid arthritis (RA) and neuropathic arthropathy or it can asymptomatically present as chondrocalcinosis (CC) on imaging [3]. Despite these common presentations, CPPD remains under-studied with a paucity of evidence-based treatment guidelines. Nomenclature issues have plagued this disease and the development of CPPD classification criteria is a necessary first step in the progression of CPPD clinical research [4•]. CPP crystal arthritis flares result in temporary but profound disability for most patients, disrupting their ability to go about day-to-day activities [5•]. In comparison with gout, there are no targeted therapies available at present for the treatment of CPPD. Therefore, therapy is aimed at ameliorating the inflammatory response and reducing the frequency and severity of CPPD flares.

BCP crystals are a group of ultra-microscopic crystalline particulates that give rise to a number of particular clinical syndromes. They are found in calcific tendinitis, rapidly destructive arthropathies such as Milwaukee Shoulder Syndrome (MSS) and more severe forms of osteoarthritis (OA) [6]. BCP collectively describes calcium phosphate crystals, including hydroxyapatite, octacalcium phosphate, tricalcium phosphate and magnesium whitlockite. Due to their small size, identification is difficult [1]. Although our understanding of BCP crystal deposition disease has improved over time, there still are no targeted therapies available for treatment. It is hoped that emergent understanding of the mechanisms through which calcium crystals mediate tissue damage will lead to the development of much needed novel management strategies for these common musculoskeletal syndromes.

This review evaluates the literature and provides updates with regard to the diagnosis and management of calcium crystal disease over the last 3 years.

### CPPD

#### Diagnosis of CPPD

CPPD was first recognised as an inflammatory arthritis in the 1960s and yet clinical research in this area has lagged behind that of other forms of arthritis including gout [7]. As a result, treatments specifically targeting CPP crystal deposition do not currently exist and many patients with CPPD suffer from inadequately treated joint pain, swelling, stiffness and ultimately, deformity [8]. Advances in CPP-related arthritis, including therapeutic trials, have been hampered by a lack of validated classification criteria, a framework that has facilitated clinical research and trials in other rheumatic diseases. It is hoped that the development of classification criteria in CPPD disease will facilitate clinical research on this common crystalline arthritis [4•].

### Role of Imaging in the Diagnosis of CPPD

Synovial fluid analysis (SFA) under direct and polarised light microscopy (PLM) is considered to be the gold standard for the diagnosis of CPPD [9]. SFA is invasive and not always possible, so imaging has a growing role in the diagnosis of CPPD. The imaging modalities commonly utilised in CPPD diagnosis include conventional x-ray and computerized tomography (CT), duel-energy CT (DECT) and ultrasound (US). Consensus-based definitions of imaging features characteristic of CPPD are an essential pre-requisite to the establishment of CPPD diagnostic criteria. An international group of rheumatologists and musculoskeletal radiologists with expertise in CPPD have recently defined imaging features characteristic of CPPD on CT, DECT and US and assembled a set of example images as a reference for future clinical research studies [10•].

The reliability and diagnostic accuracy of these new radiographic imaging definitions were assessed in a study examining patients with knee OA scheduled for knee replacement. Using the newly published definitions, two radiologists and two rheumatologists assessed the radiographic images of 67 participants for a presence or absence of CPPD in menisci, hyaline cartilage, tendons, joint capsules, or synovial membranes. The findings were compared with histologic examination of postsurgical specimens under compensated PLM. The study concluded that the new radiographic definitions of CPPD are highly specific against the gold standard of histologic diagnosis. When the described radiographic findings are present, these definitions allow for a definitive diagnosis of CPPD; however, a negative radiographic finding did not exclude the diagnosis [11].

Due to its widespread availability and low cost, plain radiography is often used as the first-line radiological investigation for the diagnosis of CPPD. Radiographic articular calcification (chondrocalcinosis) was initially identified as the cardinal manifestation of a separate disease entity “chondrocalcinosis articularis” in 1960 by Zitnan and Sitaj [12]. The updated approved definition of CPP calcification on conventional radiograph is “linear or punctate opacities in the region of fibrocartilage or hyaline articular cartilage that are distinct from denser, nummular radio- opaque deposits due to BCP deposition” [10•]. Although readily available, plain radiography is less sensitive than other imaging modalities such as US for the detection of CPPD [13].

Over the past decade, US has been extensively utilised in the diagnosis of crystal arthropathies and recent publications support its reliability as a diagnostic tool [14–16]. A systematic review concluded that although US is more sensitive and less specific than conventional radiology for identifying CPP crystals, both these techniques demonstrate great diagnostic accuracy and should be regarded as complementary to each other in the diagnostic work-up of patients with CPPD [14]. Ultrasound evidence of CPP crystal deposition in fibrocartilage or hyaline cartilage is defined as “hyperechoic deposits of variable shape and size, localized within the fibrocartilage or hyaline cartilage structure, that remain fixed or move along with the fibrocartilage/hyaline cartilage during dynamic assessment and do not create posterior shadowing” [10•]. Work from Cipolletta et al. examining 161 subjects (32 gout patients, 30 CPPD patients and 99 controls) undergoing joint aspiration further contributes that US findings had a high specificity for gout (0.92–0.96) and CPPD (0.90–0.97), while the sensitivity ranged from 0.73 to 0.85 in gout (double contour sign and tophi, respectively) and from 0.60 to 0.90 in CPPD (hyaline and fibrocartilage deposits, respectively). This study concluded that a targeted US scanning protocol of two joints bilaterally plus the target joint showed an excellent accuracy (> 90%) for the diagnosis of crystal arthritis in patients with acute mono/oligoarthritis [15].

Cipolletta et al. have further explored the spectrum of CPPD US findings at the metacarpophalangeal (MCP) joint in 60 patients with CPPD, 33 (55%) with OA and CPPD and 27 (45%) with chronic CPP crystal inflammatory arthritis. Patients underwent bilateral US examination of the MCP joints and findings were compared with age and sex-matched RA controls. CPP deposits were detected in 40% of CPPD patients and 7.5% of RA patients (*P* < 0.01). Chronic CPP crystal inflammatory arthritis demonstrated more US findings indicating CPP deposits than those with OA and CPPD. Conversely, more US evidence of structural damage was seen in OA with CPPD. This study reports alternative US patterns in different CPPD phenotypes and highlights the broad spectrum of US findings which indicate CPP deposits at the MCP joint [16]. In summary, recent publications support the accurate and reliable use of musculoskeletal (MSK) US in the diagnosis of CPPD.

DECT detects crystals based on their characteristic attenuation, described by its material-specific dual-energy gradient [17]. In addition to its ability to colour code and measure crystal deposition, DECT provides information and resolution that is equivalent to that of conventional CT. DECT is superior to conventional CT in that it can distinguish between different calcium crystals such as CPP and BCP including hydroxyapatite (HA) [18]. Given the relatively small radiation exposure of 0.5 mSv, DECT quickly rose to prominence as a non-invasive diagnostic imaging tool in crystal arthritis [19]. In a small pilot study of 10 CPPD patients with acute monoarthritis, sensitivities were 90% (62–100%) for DECT volume > 0.40 cm^3^ and 100% (74–100%) for DECT volume > 0.01 cm^3^ [20]. Access to DECT imaging can be a difficulty for some patients with suspected crystal arthritis, despite its usefulness [21].

#### CPPD Epidemiology

A recent study from the USA found that the incidence of acute CPP crystal arthritis flare was 11.4 per 100 person-years with flares being twice as common in women than in men [22]. In this study, Yates et al. analysed CPPD flares in 70 patients from a single health care system and found that recurrence occurred in approximately one-fourth of patients with acute CPP crystal arthritis and often in previously unaffected joints [22]. Subsequently, further work from this group identified other factors associated with acute flares of CPPD including OA, RA, gout, male sex, chemotherapy use, loop and thiazide diuretic use [23]. Recent updates from epidemiologic studies include data on risk in proton pump inhibitor (PPI) use and risk of associated CVD in CPPD. Hypomagnesemia is a known risk factor for CPPD, and PPI use is a known cause of hypomagnesemia [24]. A study was undertaken by Liew et al. to investigate whether PPI use is a risk factor for incident CPPD. Data for this study was derived from the UK-based IQVIA Medical Research Database. The risk of incident CPPD among PPI users versus histamine receptor 2 (H2) blocker users was derived from the database using a time-stratified propensity score-matched cohort study. No evidence was found that incident PPI use was associated with a higher risk of CPPD when compared with H2 blocker use. However, when compared with non-users, both PPI and H2 blocker users had a higher risk of incident CPPD. It is possible that H2 blocker use may have a similar causal effect to that of PPI use because users of both medications had a higher risk of CPPD than people who did not use either medication. The precision of these results is limited by the low number of CPPD cases in this study using real world data [25].

Recent epidemiological studies have suggested that CPPD disease has been associated with an elevated risk for non-fatal cardiovascular disease (CVD) events. Bashir et al. published a retrospective matched cohort analysis of the Veterans Health Administration Corporate Data Warehouse, 2010–2014 with the goal of identifying risks for cardiovascular events in patients with CPPD. This study identified 23,124 CPPD patients matched to 86,629 non-CPPD patients with > 250,000 person-years of follow-up. Although results from this work found that CPPD was not significantly associated with increased risk for major adverse cardiovascular events (MACE) (HR 0.98 [95% CI 0.94–1.02]), in models adjusted for traditional CVD risk factors, the risks of myocardial infarction, acute coronary syndrome, and stroke were significantly higher in the CPPD cohort compared to the non-CPPD cohort [26]. Subsequently, Tedeschi et al. published a cohort study examining CVD events in patients with CPP arthritis using Mass General Brigham electronic health record data. Patients with acute CPP arthritis were identified using a published machine learning algorithm with a positive predictive value of 81%. 1200 acute CPP crystal arthritis patients were matched to 3810 comparators. Acute CPP arthritis was significantly associated with increased risk for MACE in years 0–2 (HR 1.32, 95% CI 1.01 to 1.73) but not death [27].

#### CPPD Management Options

At present, there are no approved disease-modifying therapies which reduce the articular crystal deposition of CPP crystals. Currently, the goal of treatment is ameliorating the inflammatory response and reducing the frequency and severity of clinical symptoms due to CPPD. Evidence-based treatment guidelines are lacking and it is hoped that the establishment of new classification criteria in addition to clarification on defined outcome measures with the development of OMERACT CPPD Core Domain Sets will be the first step in the advancement of clinical research [28, 29]. Future development of therapies that target, inhibit, or reverse CPP crystal formation and deposition would be of great interest for all manifestations of CPPD and could have a profound impact. At present, treatments for clinical manifestations of CPPD (non-steroidal anti-inflammatory drugs [NSAIDs], colchicine and corticosteroids) are extrapolated from use in gout. Anakinra and tocilizumab can be used for treatment of CPPD in refractory cases [30, 31]. Although inconclusive, due to an underpowered study, anakinra was compared to prednisone in the treatment of acute CPPD crystal arthritis in a randomized controlled double-blinded pilot [32]. This study demonstrated similar effectiveness between anakinra and prednisolone in the treatment of acute CPPD arthritis with anakinra seeming to have a faster onset of action than prednisone. Although the trial was not completed due to poor recruitment, the data could be helpful in designing a larger multicentre trial in the future.

#### The Effect of Pathogenic Crystals on the Production of Cytokines

Calcium crystals are able to induce an inflammatory response resulting in the production of IL-1β after NF-Kb activation and through the NLRP3 inflammasome [33]. An important work by Scanu et al. aimed to further investigate the in vitro inflammatory response induced by MSU, CPP and BCP crystals in three distinct leukocyte populations, polymorphonuclear cells (PMN), monocytes and lymphocytes [34]. Cells isolated from healthy volunteer blood were stimulated for different time periods with increasing MSU, CPP or BCP crystal concentrations. IL-1β, IL-8, IL-6, CCL2, IL-1Ra and TGFβ1 levels were determined by ELISA. Although with limitations (this study did not explore upstream or downstream pathways), the results demonstrated that the three types of leukocytes from the same donor behave differently after stimulation with crystals. CPP crystals were the most potent stimulants of inflammatory cytokines. Better understanding of these inflammatory pathways stimulated by crystals will hopefully pave the way for future targeted therapies [34].

#### CPPD Potential Novel Treatments

There has been evolvement in the role of histone deacetylase inhibitors (HDACis) which have been shown to downregulate CPP crystal formation in human articular chondrocytes [35]. Extracellular pyrophosphate (ePPi), calcium, and extracellular matrix (ECM) are essential components of CPP crystal formation [36]. A high concentration of ePPi in synovial fluid is positively correlated with the formation of CPP crystals. HDACis were able to decrease ePPi and CPP formation by regulating ankylosis human (ANKH), ectonucleotide pyrophosphatase 1 (ENPP1), and tissue non-specific alkaline phosphatase (TNAP) expressions in human primary cultured chondrocytes. The underlying molecular mechanisms of HDACis-mediated regulation of ANKH, ENPP1, and TNAP expression might be associated with the histone acetylation status of the promoters of these three genes. HDACIs may have the potential to be developed into drugs to prevent CPP formation or treat CPP-related diseases in the future.

#### Updates in Surgical Management of Patients with CPPD

Until recently, unicompartmental knee arthroplasty (UKA) was not recommended in patients with CPPD, limiting surgical options for these patients [37]. The rationale was that the remaining cartilage might trigger further production of CCP crystals resulting in inferior outcome. A systematic review was undertaken by Moret et al. to analyse and compare outcomes including progression of OA and prosthesis survivorship after UKA or total knee arthroplasty (TKA) in patients with CC versus patients without CC. A total of 3718 patient knees were included in 8 publications, at the time of surgery, the mean age was 69 years and the prevalence of CC ranged from 12.6 to 36%. CC was considered to be present if there were either radiologically visible calcifications within the soft tissues or the cartilaginous structures preoperatively or CCP crystals seen histologically in tissue samples or in synovial fluid by PLM. The presence of CC did not significantly influence functional and clinical outcome, implant survival or radiologic progression after either UKA or TKA. This study suggests that CC may carry less clinical relevance in the context of those patients suitable for arthroplasty [38].

In addition to this, a recent cross-sectional study using the US National Inpatient Sample database was published identifying CCP and non-CPPD patients who underwent TKA from 2006 to 2014 [39]. This study aimed to assess predictors and outcomes of TKA in patients with CPPD. The data collected included patient demographics and comorbidities, outcomes following TKA including in-hospital mortality, complications, length of hospitalization, hospital charges and disposition. Among the 5,564,005 cases that underwent TKA, 0.2% had CPPD with a median age of 72 and 53.7% were female. Compared with non-CPPD patients, CPPD patients were more likely to be older (mean 72 vs 66 years; *p* < 0.001) male and white. CPPD patients were more likely to have > 2 co-morbidities and discharged to an inpatient/rehabilitation facility rather than home. Compared to non-CPPD patients, TKA in those with CPPD was associated with greater length of hospital stay. Hospital complications such as myocardial infarction and reoperation were more frequent in CPPD patients [39]. Therefore, although Moret et al. suggest the presence of CC, it does not affect the indication for arthroplasty, this subsequent study by Parperis et al. highlights some of the post-operative challenges that this multimorbid cohort face.

## BCP Crystal Deposition Disease

### Diagnosis of BCP Crystal Deposition Disease

A major challenge to the diagnosis of BCP crystal deposition disease is that there are no simple bed-side tests to identify their presence, in contrast with CCP or MSU crystals [29]. BCP crystals cannot be visualized using PLM. Specialized testing such as Alizarin red S staining or transmission electron microscopy can identify BCP crystals in synovial fluid from affected patients but are not routinely available in clinical practice [40]. It is hoped that with the further development of sensitive imaging techniques including US, the diagnostic yield will be improved.

Although validated definitions for MSU and CPP deposition on US imaging have been released by OMERACT, this has not yet been the case for BCP crystal deposition [10•, 41]. US is routinely used in the diagnosis of calcific tendonitis/periarthritis but its reliability and validity have yet to be assessed in hydroxyapatite (HA) detection. Filippou et al. completed a proof of concept study with the aim of investigating the US attenuation characteristics of CPP, MSU and HA crystals [42]. Sixteen synthetic crystal suspensions with known concentrations of each crystal were analysed. The CPP crystal suspensions did not generate attenuation or acoustic shadowing of the beam in comparison with HA and MSU suspensions which substantially attenuated the beam and generated acoustic shadowing from a different given crystal concentration. The study highlighted the potential ability of US to distinguish between these different crystals based on their variable US appearance and holds potential to enhance the diagnostic performance of US in crystal arthritis including BCP deposition disease [42].

### Treatment of BCP Crystal Deposition Disease

Treatments for BCP-related arthritis are generally not evidence-based and few comparative effectiveness trials exist. First-line therapies for BCP-related arthritis include NSAIDs and intralesional corticosteroids. Large calcific densities associated with chronic symptoms are often managed with a variety of interventions designed to break up the mineral deposits [6]. Although there have been no novel therapies targeting BCP deposition in recent years, there has been some progress in understanding the clinical presentation and indeed the role of BCP crystals in OA. The characterization of novel mechanisms of BCP crystal formation and resultant tissue damage should ultimately lead to more effective treatment strategies for these syndromes. We will now summarise some of the key updates.

### Fetuin-A

The use of a potent calcium-binding peptide derived from fetuin-A has been shown to counteract crystal deposition in OA. Van den Akker et al. demonstrated that the cyclic-inverso fetuin-A-derived peptide inhibited calcification in human articular chondrocytes from patients with end stage OA undergoing knee replacement [43•]. This molecule was also found to be effective in a vascular smooth muscle cell calcification model and during osteogenic differentiation of bone marrow–derived stromal cells. Intra-articular injection of the cyclic-inverso peptide reduced cartilage degeneration and improved mobility in Lewis rats with OA. The cyclic-inverso AHSG 1–30 peptide holds the potential for future application in OA. However, further studies are required in alternative calcification models [43•].

### Evolution of Cartilage Calcification in OA

BCP-based calcification of cartilage is a common finding in OA and is often linked with severity of disease. Calcium crystallites are identified in most affected joints, and the presence of these crystallites is closely correlated with the extent of joint destruction [44]. To date, little has been known about the evolution of cartilage calcification during OA development. A recent paper investigated the stage and location-specific characteristics of minerals in human OA cartilages using multiple nano-analytical technologies [45•]. Investigators found that OA progressed by both top-down calcification at the joint surface and bottom-up calcification at the osteochondral interface. The top-down calcification process started with spherical mineral particle formation in the joint surface during early stage OA followed by fibre formation and densely packed material transformation deep into the cartilage during advanced-stage OA. The bottom-up calcification started in early-stage OA when an excessive layer of calcified tissue formed above the original calcified cartilage, exhibiting a calcified sandwich structure. Over time, the original and upper layers of calcified cartilage fused, which thickened the calcified cartilage region and disrupted the cartilage structure. The mineral crystals in the joint surface cartilage and osteochondral interface region show different mineral transformation pathways, associated with the different biological environments where they form and assisted by the nearby transdifferentiated chondrocytes. The overarching aim of the authors was to further understand the mechanism of OA progression with the aim of targeting cartilage pathological calcification in the treatment of OA in the future.

### BCP Crystals in Tenocytes

In addition to cartilage calcification, BCP crystals contribute to several syndromes associated with tendon disease such as MSS. Tenocytes are stromal cells found within tendons where they maintain and repair tendon extracellular matrix by the regulated expression of matrix proteins, matrix degrading enzymes and their inhibitors [46]. Dysregulated expression of these matrix proteins and enzymes may contribute to tendon degeneration. BCP crystals are frequently deposited within tendon structure and are likely to be in contact with tenocytes. While numerous studies have demonstrated that BCP crystals interact with immune cells and other stromal cells leading to altered expression of interleukins, prostaglandins and metalloproteinases, little is known about the interactions between BCP crystals and tenocytes and how this contributes to these clinical syndromes. These interactions were further studied by Chhana et al. where in vitro assays, Elisa and PCR were used to assess changes in human tenocytes cultured with BCP crystals [47]. They found that BCP crystals induce extracellular matrix remodelling enzymes including prostaglandin E_2_ and MMP-1 protein but did not induce inflammatory cytokines in tenocytes. These findings suggest that BCP crystals do not have major inflammatory effects on tenocytes, however BCP crystals are likely to interact with tenocytes to reduce tendon matrix integrity, which may contribute to tendon damage in tendinopathy-related conditions such as MSS.

Hopefully, this recent work will form a basis for further research and lead to development of targeted therapies in this somewhat neglected area.

## Future Directions and Conclusion

Calcium-crystal deposition diseases are likely to continue to increase in incidence and prevalence as populations age and will continue to associate with a high burden of disability. Therefore, this is an area of research of great potential importance. There has been some progress in understanding the mechanisms of disease in the last 3 years but novel therapies targeting crystal deposition remain elusive. Significant progress has been made in the area of diagnosis with improvements in the use of non-invasive imaging including US. There is substantial scope for further investigation into the mechanistic and genetic pathways behind these diseases. The newly drafted ACR/EULAR classification criteria for CPPD will be published shortly. They have excellent performance characteristics and will facilitate research in this important field. In addition, the establishment of the Gout, Hyperuricemia, and Crystal-associated Disease Network (G-CAN) has been of great impact in increasing research and collaboration in crystal deposition diseases.
